# A qualitative exploratory study of teachers’ perceptions of movement behavior of students with intellectual disabilities in the school context

**DOI:** 10.3389/fspor.2025.1655758

**Published:** 2025-11-04

**Authors:** Tim Messerschmidt, Nadja Schott

**Affiliations:** Department for Sport Psychology and Human Movement Science, Institute of Sport and Movement Science, University of Stuttgart, Stuttgart, Germany

**Keywords:** developmental disorders, individual, task, environmental constraints, qualitative interview, motor skills, Newells's constraint model, gentile taxonomy of motor skills

## Abstract

**Introduction:**

This study explored teachers’ perceptions of movement behavior of students with intellectual disabilities (ID), intending to support the development of adapted motor assessment instruments and interventions.

**Methods:**

The study involved sixteen teachers from German schools for children and adolescents with ID, each of whom worked as a teacher with a specific focus and background and was similarly involved in the school's daily routine. A semi-structured interview focusing on six central research questions was conducted with each teacher who participated. The interviews were audio-recorded, transcribed, and analyzed using qualitative text analysis with the help of MAXQDA.

**Results:**

Six main thematic codes were generated within the analysis. Teachers’ perceptions revealed that students show different levels of motor development and that most demonstrate potential for motor learning. As ascertained by the teachers, students can generally be considered a remarkably heterogeneous population, encompassing individuals with diverse diagnoses, needs, and motor and cognitive abilities. The teachers also reported that the students are generally able to perform simple everyday activities and are keen to learn or perform activities that help them become more independent and self-determined. They also mentioned that they have difficulties performing fine and gross motor activities. A range of individual, task-related, and environmental factors influences movement behavior and desires as well as problems related to movement behavior. Moreover, various sports and movement activities, as well as different forms of therapy, are offered at German special schools for students with ID. In the context of teaching practices, the teachers underlined the importance of using an individualized and gradual methodological approach to promote students’ movement behavior.

**Discussion and conclusions:**

The findings suggest that both motor interventions and assessments should be adapted to the specific needs or characteristics of children and adolescents with ID so that they can participate and perform. Moreover, motor assessments and interventions should be systematically structured to address the specific needs of children and adolescents with ID, and to optimize the progress in motor learning. Within physical training, practitioners should place value on improving activities that are important for increasing independence and mastering everyday life, as well as for the overall personal development of the individual with ID. Attractive and promising tasks as well as assistive technology may be regarded as appropriate means to promote movement behavior. Future research should include the views of students and their families and other exploration techniques, e.g., direct observation or group interviews, to expand the understanding of movement behavior.

## 1 Introduction

From a biomedical perspective, intellectual disability (ID) is a lifelong condition characterized by a significant impairment in intellectual functioning and adaptive behavior that originates in the early developmental period (1–3). According to the Diagnostic and Statistical Manual of Mental Disorders, 5th Edition (DSM-5), published by the American Psychiatric Association (APA) (2), a diagnosis of ID requires an individual to show significantly below-average deficits (2 or more standard deviations) in intellectual functioning and/or adaptive functioning confirmed using standardized tests or clinical evaluation during the developmental period (2). The degree of impairment in intellectual functioning and/or adaptive behavior, as determined by standardized testing or clinical assessment, determines the severity of an ID. The DSM-5 classifies ID into four degrees of severity: mild, moderate, severe, and profound. The International Classification of Diseases, 11th Edition (ICD-11), published by the Word Health Organization (3), categorizes ID similarly, with the addition of a fifth category, “provisional” and sixth category “unspecified”.

Typical diagnoses, clinical pictures, or syndromes associated with ID and caused by intrinsic or extrinsic factors include Down syndrome (DS), Fragile X-Chromosome Syndrome, or fetal alcohol syndrome (2). Individuals with ID often experience illnesses, disorders, or health problems concurrently. These include Autism Spectrum Disorder (ASD), epilepsy, childhood cerebral palsy, malformations, and behavioral problems (2). From an etiological and diagnostic perspective, there are differences between ID, ID combined with other diagnoses, and other (mental) developmental disabilities such as Borderline Intellectual Functioning (BIF) and ASD (2, 3). However, numerous symptomatic similarities can be observed in individuals with these disorders, including difficulties in social interaction, communication problems, behavioral problems, and restrictive (repetitive) behaviors (2, 4, 5).

The diverse symptoms of ID and other (neuro)developmental disabilities can result in various needs for promotion and support in people with these disorders. Children with ID or other developmental disabilities often attend special education schools to meet their specific needs and receive appropriate support. Schools of this type usually specialize in particular academic or promoting fields and pursue a specific educational and upbringing mission based on the students' individual requirements, opportunities, and needs. The symptoms, signs, or effects of ID are remarkably diverse. ID often manifests through deficits in intellectual functioning, such as learning and problem-solving, or impairments in adaptive functioning involving many social and practical skills, such as communication, organizing, and independent living (1, 2). In addition to cognitive and psycho-social limitations, ID is often accompanied by motor impairments (6, 7). Object control skills, such as catching and throwing, and locomotion skills, such as balancing and jumping, are frequently impaired in individuals with ID (8, 9). Additionally, children with ID show lower levels of physical fitness (10) and physical activity (11) than their typically developing (TD) counterparts.

Many studies have examined the motor performance of school-aged children and adolescents with ID on fundamental movement skills (FMS) and compared it to that of TD children. This has been done using standardized and norm-referenced diagnostic tools such as the Movement Assessment Battery 2 (MABC-2) (12), the Test of Gross Motor Development 2 (TGMD-2) (13), and the Bruininks-Oseretsky Test of Motor Proficiency 2 (BOTMP-2) (14). Studies have indicated that school-aged children and adolescents with ID exhibit poorer motor performance on FMS than their TD peers. Significant differences were found between children with (mild) ID and TD children on both TGMD-2 subtests (locomotion and object control skills) (8, 9, 15–18). Children with (mild) ID performed significantly lower than the TD children, with large effect sizes (8, 9). Moreover, it has been found that children with ID score significantly lower on object control tasks than on locomotion tasks in the TGMD-2 (8, 15). This suggests that they may encounter significant challenges in tasks requiring upper-limb motor coordination.

Furthermore, studies have investigated the motor performance of children and adolescents with ID and/or other mental developmental disabilities and TD children on FMS and compared it with each other. Lower FMS performance compared to their TD peers has also been found in school-aged children with intellectual and/or mental developmental disabilities, including children with DS (19–21), children with ASD (5, 22, 23), and children with BIF (9). Significant differences were observed between children with both DS and BIF and TD children on both locomotor and object control skills (TGMD-2 subtests), with effect sizes being large for children with DS (21) and moderate-to-large for children with BIF (9). Children and adolescents with ASD performed significantly lower on the total MABC-2 test score, aiming and catching, and balancing (components of MABC-2) than their TD peers, with medium effect sizes (23).

Moreover, numerous studies have attempted to investigate the relationship between cognitive and motor function in school-aged cognitively impaired children with varying degrees of severity, including moderate and mild ID and BIF. The studies have shown an association between cognitive and motor function, with children with lower IQs showing poorer BOTMP-2, MABC, or TGMD-2 FMS performance than those with higher IQs (5, 9, 16, 24).

A closer examination of the literature reveals that most studies investigating FMS in children and adolescents with ID are quantitative and test-based, while the views of teachers, children, or their families have rarely been directly involved. Assessment of FMS in previous research with children and adolescents with ID has been based primarily on a bottom-up or performance-based approach using standardized and norm-referenced diagnostic instruments. However, using these instruments to measure motor skill performance in children with ID may be associated with various problems and may be inappropriate for them. This can be explained by the fact that these instruments were not explicitly developed for this population. The use of these instruments may be problematic due to the discrepancy between the demands of the movement tasks, the assessment requirements (instructions), and the individual preconditions of many children and adolescents with ID and other developmental disabilities. Children and adolescents with ID often have short attention spans and poor memory, as well as other cognitive, psycho-social, and (partly) physical-motor limitations (2, 4).

Consequently, they may have difficulty performing or understanding some movement tasks. As a result, they may not be able to show their capabilities or demonstrate their actual motor performance. In addition, some of the items or movement tasks included in the instruments are not of high practical relevance for children and adolescents with ID. These encompass locomotion skills, such as horizontal jumping, hopping, and galloping, as well as object control skills, such as overhand throwing for distance or placing pegs in a pegboard, that are rarely encountered in daily life. It may be speculated that performances on such movement tasks may be influenced by cognitive rather than motor skills of children with ID. To increase the ecological validity of motor assessments in relation to children and adolescents with ID, the focus should be on movement tasks that are directly important for everyday functioning and self-care and should therefore include activities, such as grasping and placing objects, climbing stairs, putting on shoes, and other similar tasks. Improvements in such tasks may have positive effects on daily functioning and independence. In addition, movement tasks should be generally adapted to reduce their demand by using larger or more easily controllable objects and considering visual, verbal, or physical support in the context of assessments.

In light of the above challenges and the development of practical assessment tools, it is critical to understand the unique abilities, limitations, and preferences of children and adolescents with ID. An in-depth qualitative study approach can facilitate this understanding and provide an important foundation for developing tailored quantitative motor skill assessments and interventions to promote motor skill development. Appropriate approaches for motor skill assessment are crucial, as they can help to describe the psychomotor profile of students with intellectual disabilities precisely. Moreover, adapted interventions that allow students to learn and execute motor skills and to increase motor skill performance may be essential, as these can contribute to a range of health and performance benefits, including improved physical fitness and cognitive functioning, greater independence and self-determination, and a more active lifestyle (4). Given the diverse experiences that special education teachers have with the movement behaviors of children and adolescents with disabilities, it can be assumed that they are well-positioned to provide insight into this topic.

In view of the fact that previous research was primarily based on inappropriate quantitative and test-based assessments and lacked teachers' views, this study aimed to explore teachers' perceptions of movement behavior of students with ID to support the development of adapted motor assessments and interventions, and thus guide inclusive curricula, classroom adaptations, and health promotion. A dual conceptual framework combining Newell's constraints model (25) with Gentile's taxonomy of motor skills (26) was adopted, guiding our analysis. This framework offers a structured approach to analyze movement behavior based on environmental, task-related, and individual constraints, as well as to classify motor skills depending on movement execution and environmental context. Its application may be of particular importance within the context of motor assessments and interventions in physical education of children with ID, as it provides a structured approach to describe, explain, and promote movement behavior based on the individual characteristics of the individual and the demands of certain activities. Based on this framework, the study addressed the following main research questions:

- Research Question (RQ) 1: What observations can be made regarding students’ movement behavior in the school context, e.g., during classes in the classroom or in the gym, or during recess in the school building or the schoolyard?
- RQ 2: Which movements or physical activities in everyday life or in the movement context can the students perform independently and successfully, only with difficulty, or not independently and successfully, or do the students want to learn or perform?
- RQ 3: What exactly is the problem with the movements or physical activities in which the students encounter challenges during execution?
- RQ 4: What factors could potentially influence the execution of everyday and athletic movements, motor performance, and physical-motor goals or desires?
- RQ 5: What physical activities are typically taught in physical or physical-motor education classes?
- RQ 6: What are the key considerations when teaching physical activities (skills, sports) to students with ID?

## 2 Methods

### 2.1 Philosophical assumptions and research approach

The authors brought certain philosophical assumptions or beliefs to the research process. As the study aimed to interpret subjective perceptions or experiences described in the interviews, this study was based on a constructivist (interpretivist) research paradigm. This comprises relativistic ontology, subjectivist epistemology, and a hermeneutic (qualitative) methodology (27). Consequently, the assumption was made that reality depends on the observer, meaning that multiple forms of reality exist and knowledge is personal or subjective. Furthermore, it was thought that the research process is seen as an interaction process in which the researcher and the object change, resulting in knowledge or knowledge acquisition. In addition to philosophical assumptions and interpretive frameworks, the researchers were aware of their perspectives and experiences brought to the research process (27). They knew their characteristics were embedded in the research process and outcome. In line with the research paradigm, the study used a research approach to explore or analyze children and adolescents based on teachers' perceptions, considering hermeneutics (or dialectics). The basic assumption was that studying a particular group of people is possible based on other people's perceptions or experiences. Moreover, as the subject of the study has not yet been adequately explored in previous research, it was deemed essential to investigate the research subject in an exploratory manner using a qualitative and inductive research approach.

Methodologically, this research commenced with a comprehensive literature search on motor development and assessment in children with ID. The study's aims and methodological approach were determined based on the strengths and weaknesses of previous research. Since several different topics were of interest or intended to be explored, an interview guide for a semi-structured interview was developed. If necessary, the developed interview guide was tested and modified in a subsequent pilot phase. The pilot phase was also used to train the interviewer. The data collection and analysis phase was initiated after the planning, decision-making, and preparation phases were completed. In this research phase, a one-off semi-structured interview was conducted with each participant at a location and time convenient to them. A few days after the interview, the participant was allowed to revise or add to the interview transcript prepared by the interviewer. This enabled the interviewee to respond more flexibly to the experiences gained, which can contribute to enriching the results.

Study sampling was based on data saturation. From the 14th interview onwards and in two further interviews, it was determined (with the help of the prepared case summaries of each interview conducted) that the reported content from the interviews was remarkably similar and that no new, central information was added. For this reason, the decision was made that data saturation had been reached. This method was employed to ascertain data saturation before forming categories (28), constituting a component of the evaluation process. Data saturation was evaluated through ongoing discussions among the research team during the data collection. To ensure rigor, the research team continuously compared the content of each interview with the preceding ones, based on a qualitative approach.

All interviews were conducted and evaluated by the first author, who possessed a background in sports science and a specific understanding of the topic under investigation. He also had professional experience as a physical education teacher and therapist in the special education context. Throughout the research process, the first and second authors engaged in continuous meetings to discuss and reflect on the findings to enhance the research's rigor and quality. The ethics committee of the University of Stuttgart approved this study (protocol number 2023-51), for which an application for a statement on the research project was submitted.

### 2.2 Participants

In order to provide a comprehensive and nuanced understanding of the research topics, teachers with diverse backgrounds and expertise were recruited for the exploration. These teachers represented different age groups, work focuses, and schools, including those serving children and young people with ID aged between 7 and 18. The initial recruitment of teachers was conducted by the first author, who selected individuals deemed well-suited to answering the research questions or who could contribute to the findings. Efforts were made to ensure that the number of teachers with similar work foci was approximately equal. Teachers must be fully qualified educators at educational institutions for children and young people with disabilities. They were contacted directly or via written correspondence to ascertain their interest in participating in the study. Those interested in participating were subsequently informed about the study's aim and procedure during a personal or telephone interview. Considering the data saturation model for sampling, 16 teachers participated in the study (13 women and three men). The participants' ages ranged from 29 to 61 years, and their work experience ranged from 2 to 32 years. The sample consisted of six special education teachers (SETs), one special education teacher (SET) with a focus on intellectual development (an educator with additional training), three SETs with a focus on physical and motor development (physiotherapists or occupational therapists with further training), one educator, four physiotherapists, and one occupational therapist. One-half of the participants focused on mental development, while the other half focused on physical and motor development. The sample included five participants in age groups 6–11 and 11–16 years and six participants aged 16–20.

Regardless of the educational background, each participant was similarly involved as a teacher in the school's daily routine and, as part of the teaching duties, worked with students in regular classes in the classroom or in physical education classes in the gym, and supervised them during recess in the schoolyard. In addition to classroom-integrated therapy, teachers with a therapeutic background sometimes worked with the students within additional individual therapy sessions in therapy rooms.

Prior to the interview, each participant was informed verbally and in writing about the purpose and procedure of the study, as well as the general conditions. They were also informed that they could withdraw from the study at any time without giving reasons and without any disadvantages. Participation was voluntary and unpaid. They also understood that their data would be kept confidential and anonymous. Finally, they were asked to sign an informed consent form and a privacy statement.

### 2.3 Data collection

The interviewer interviewed each participant at a location and time that was convenient for them, such as their place of employment or their home. Prior to the interview, each participant was informed about the study procedure and provided written informed consent. They were then asked to complete a specially developed interview questionnaire to collect personal information, including age, sex, occupation, and work experience in special education. This was completed immediately before the interview. Occupational data included the focus of the participant's work in special education and the age range of the students in the context of their employment at the school.

Upon completion of the questionnaire, a personal, semi-structured interview was conducted with each participant. The opening section of the interview included general questions designed to introduce the topic and determine the importance of the topic to the individual. The main section of the interview began with the introduction of the study's central question, which served as a narrative stimulus. This question is related to the teachers' perceptions of the movement behaviors of students with ID in their daily school activities. These aspects were further explored in the subsequent interview through *ad hoc* questions. In addition, questions from the interview guide were used to examine the above aspects further. Depending on the interview situation, the order of the questions may have been changed or skipped, as they may have already been addressed. In the case of short narratives triggered by the narrative stimulus, the interview guide was used immediately to guide the conversation. The interview guide comprised 11 questions and was structured according to thematic focus (see Supplementary Material). The main questions asked in each interview were the aforementioned research questions.

At the end of the interview, the most critical aspects reported or discussed were summarized, and the interviewees were allowed to supplement the elements mentioned or add other aspects. Each interview lasted approximately 45 min (range 30–65 min). A follow-up interview was then conducted to discuss the interview situation or issues raised in greater depth. A postscript was written, which could be used to interpret the data and, if necessary, to exclude interviewees from the evaluation process (29). This document included notes on the behavior of the participant and the interviewer and comments on the general atmosphere of the interview. Each interview was recorded with an audio recorder [smartphone OPPO A74 (CPH2219)] and later transcribed using various methods. After the transcription was completed, the interview transcript was presented to the interviewee (in writing) with the request to make any necessary additions or corrections. Two participants made additions.

All interviews were conducted in German. For publication, quotations were translated into English by the first author. While ellipses replaced a sentence's inconsiderable parts (fillers, etc.), important attributes were included in parentheses to maintain contextual meaning. A second bilingual researcher checked the translations to ensure accuracy and clarity.

### 2.4 Data analysis

#### 2.4.1 Data preparation

The recorded audio-based interview data were prepared for subsequent analysis using successive and simultaneous steps (30). Each recording was transcribed directly into written form (full text) without transcription software. As the data preparation focused on the content and thematic level, the recorded interviews were transcribed according to the simple transcription rules proposed by Kuckartz (30). Personal data was anonymized or pseudonymized, so it was impossible to identify the respondents. Finally, the interview texts were formatted according to specific rules (30) using Microsoft Word (Office 365) and imported to the software used for subsequent analysis. A detailed description of the regulations considered for transcription, anonymization, and formatting can be found in Kuckartz (30).

Quotations were used to illustrate the themes or central contents identified. Selection criteria included expressive clarity and diversity in terms of different schools and professional profiles. To avoid overemphasizing particular participants, quotations were distributed across different participants.

#### 2.4.2 Data evaluation

The transcribed text-based data material was analyzed using content-structured qualitative text analysis (30). This analysis was used to extract the main contents, themes, or aspects from the interview texts (based on a deductively and/or inductively developed code system).

First, an initial text work was done in which all cases (interview texts) were carefully read, important passages were marked, and notes were taken. This step was finalized by writing a summary of each case and creating an overview of all case summaries. Then, the main thematic codes were developed based on the interview guide, i.e., the code development was done deductively. Passages describing motor skills were coded deductively based on Gentile's taxonomy. This taxonomy represents a two-dimensional classification system that allows motor skills to be categorized according to the variability of the environmental context (stationary or regulatory conditions without or with intertrial variability) and the variability of the movement execution (no body movement or body movement without or with object manipulation) and comprises a total of 16 motor skill categories (26). Therefore, according to this taxonomy, passages describing motor skills performed in a static environment were assigned to the main thematic code “motor skills in a static environment”. Similarly, passages describing motor skills as being performed in a dynamic environment were assigned to the main thematic code “motor skills in a dynamic environment”. Passages describing problems related to movement behavior or factors influencing movement behavior were coded deductively based on Newell's model (25). In cases of doubt, the assignment of text passages to codes was based on the overall assessment of the text and was ultimately based on the author's interpretation (see Supplementary Material).

As part of an initial run-through of a portion of the material [approx. 10%–25% of the entire evaluation material as proposed by Kuckartz (30)], the thematic codes and subcodes developed *a priori*, and their definitions were tested for applicability to the empirical material. Themes not expected to come to the fore were supplemented with thematic codes in the code system as part of this test run. Thus, codes were also developed inductively in this step. The entire material was coded with the main codes in the next step. Text passages that matched the content or were relevant to the research question were assigned to the codes. Codes and subcodes were then determined inductively from the material after all text passages assigned to a code had been compiled. Finally, the entire material was coded using the generated differentiated code system. All analytical steps conducted within the text analysis were performed using MAXQDA (Version 2022). Examples of coded passages and their corresponding codes can be found in the supplementary material (see Supplementary Table 7).

### 2.5 Quality of the study

The principles of qualitative thinking and procedures described by Mayring (31) and the qualitative quality criteria proposed by Mayring (32) and Kuckartz (30) were considered when planning the study and collecting and analyzing the data to ensure the quality of the study. Particular attention was paid to the openness within the interview and a systematic step-by-step analysis of the text-based data using a theory-guided code system developed from the material within the framework of qualitative content analysis (31). Considering the relativistic ontological or subjective epistemological position adopted, each interview was conducted with equal respect and evaluated based on rules and the notes taken during and after the interviews. The focus was on quality rather than quantity, i.e., the codes were equally important regardless of the number of codings. To increase the reliability of the coding or to facilitate the application of the codes developed during the evaluation process, a coding guide was created, containing a definition, an example, and a coding rule for each code developed. Furthermore, to ensure the quality of the coding process, a qualitative approach through joint review of the codes was chosen at the beginning of the coding phase. In the course of this, the first and second author applied the codes created by the first author to critical text passages (selected by the first author) of several cases, considering the developed coding guide. Codes that were ambiguous in application were defined more precisely, merged with other codes, or removed if necessary. As mentioned above, the code system was further developed and improved through regular meetings between the first and second authors during the data analysis phase to adequately represent the information reported in the interviews and to draw high-quality conclusions. The inclusion of teachers from different schools for children and adolescents with ID and with different focuses of work, combined with the sampling method aimed at data saturation, means that it can be assumed that a wide range of information was collected and that the findings generated are of great significance. Spending time directly in the research field during the research process allowed the first author to compare the teachers' perceptions with his perceptions and to discuss the findings with the teachers, which helped to validate the findings and increase the generalizability and transferability of the findings generated.

## 3 Results and discussion

The study's empirical findings, generated based on evaluating the processed text-based data using qualitative content analysis according to Kuckartz (30), are presented below. The content analysis yielded six principal thematic codes. The principal codes are as follows: (a) *general characteristics in children and adolescents with ID,* (b) *motor skills mastered independently, performed with difficulty and wished to be learned by students with ID*, (c) *problems related to movement behavior in students with ID*, (d) *factors influencing movement behavior and movement desires of students with ID*, (e) *content related to physical-motor and therapeutic activities in schools for students with ID,* and (f) *teaching approaches within sports, movement, and therapeutic activities in schools for students with ID*. Table 1 presents the main codes generated in the analysis with brief descriptions and examples. The supplementary material includes the generated code system in tabular form to clarify further and enhance the data obtained. The final differentiated code system developed can be obtained from the first author upon request.

**Table 1:** Overview of main codes generated within the analysis with brief descriptions and examples.

Main code	Code description	Example
General characteristics of children and adolescents with ID (*n* = 479)	General physical-motor, psycho-social, and cognitive characteristics in children and adolescents with ID, as reported by the teachers	Physical-motor characteristics (fine motor problems, overweight), psychological characteristics (low self-esteem), etc.
Motor skills mastered independently, performed with difficulty, and were wished to be learned by students with ID (*n* = 493)	Motor skills mastered independently, performed with difficulty, and wished to be learned by students with ID, as reported by the teachers	Eating, swinging, catching, kicking, riding a bicycle, etc.
Problems related to movement behavior in students with ID (*n* = 234)	Problems (individual, task-related, environmental) related to movement behavior in students with ID, as mentioned or assumed by the teachers	Physical-motor problems (strength deficits), psychological problems (fear of heights), task-related problems (complex movement tasks), etc.
Factors influencing movement behavior and movement desires of students with ID (*n* = 404)	External (socio-ecological) and internal (physical-motor, psychosocial, and cognitive) factors influencing movement behavior (motor development, motor learning, motor control) and movement desires of students with ID, as mentioned or assumed by the teachers	Physical-motor factors (level of motor development), psychological factors (motivation), task-related factors (group-specific exercise program), etc.
Content related to physical-motor and therapeutic activities in schools for students with ID (*n* = 261)	Content about physical-motor and therapeutic activities in schools for students with ID, as reported by the teachers	Sports activities (running, hopping, bouncing a ball), forms of therapy (physiotherapy, speech therapy), therapeutic measures (gait training), etc.
Teaching approaches within sports, movement, and therapeutic activities in schools for students with ID (*n* = 161)	Teaching approaches within sports, movement, and therapeutic activities at schools for students with ID, as reported by the teachers	Individualized approach, gradual methodological approach, brief verbal instructions, etc.

Numbers in brackets indicate the number of codings.

### 3.1 General characteristics in children and adolescents with ID

In the interviews, teachers reported various characteristics of students with ID related to their body, motor function, psyche, social behavior, and cognition. The teachers' perception revealed that students often show physical (“scoliosis”, “hypertony”, “hypotony”, “overweight”), motor (“motor developmental delay”, “impaired motor functions”), and psycho-social impairments (“… you notice very clearly that there is a great deal of insecurity at the beginning, especially in relation to the other children, and this quickly leads to comparisons.” [participant 3 (P3), SET], “problems with group activities” (P12, SET)) besides cognitive impairments [“…  for many of those, it is simply not possible to understand the movement task …” (P1, SET)]. Overall, teachers believe that students with ID constitute a highly heterogeneous population, encompassing individuals with diverse diagnoses, symptoms, needs, motor abilities, cognitive abilities, and performance, as illustrated in the following quote: “Basically, there are vast differences, ranging from students with severe multiple disabilities and massively restricted mobility to those who are relatively physically independent, active, and functional to the extent that everyday life works well for them.” (P9, physiotherapist). This is corroborated by the findings of Kannewischer and Wagner (33), who examined this population at schools for students with ID and found that different diagnoses or impairments occur within this population.

The teachers indicated that developmental delays, DS, ASD, epilepsy, hypertension, and hypotension were common in students with ID. It was highlighted that hypotension (“I am thinking of children with DS, who often simply have low muscle tone.” (P13, SET) as well as hypermobility [“… students with DS are usually more mobile than immobile.” (P1, SET)], are prevalent traits among students with DS. Moreover, teachers explained that motor deficits or delays are often observed in early childhood (kindergarten age) and frequently persist into adolescence (“…  we're happy in kindergarten when three-year-olds have finally reached the developmental level of a one-year-old. …  Unfortunately, development often stagnates when the children get older. … if it's about differentiation, …  playing badminton, …  sense of rhythm, …  then you quickly notice that children with  …  psychomotor retardation can't keep up with . ..  their peers (those with normal development).” (P5, physiotherapist). Most students exhibited impaired fine and gross motor functions and reduced physical fitness, as reported by the teaching staff: “…  there are children who simply have fine motor skills difficulties—(with) eating, writing, doing puzzles  …  (P13, SET)”, “…  they had  …  motor skills difficulties, whether in gross motor skills such as walking and running, or in fine motor skills, …” (P6, SET), and “… because our students, due to their physical condition, often get out of breath quickly, often have little power  …” (P1, SET). Furthermore, as explained by the teachers, motor performance in individuals with ID often stagnates or deteriorates during puberty: “…  I often see that, as puberty approaches, they often gain weight,  …  and their motor development stagnates and regresses.” (P10, SET), “…  when they reach puberty, it is often the case that  …  they go steps back again, that one can achieve development up to that point, and then one has to make sure that it is maintained  …” (P15, physiotherapist). In general, the teachers emphasized that children with ID can make significant progress in learning during the early years (“I have worked for many years, among other things, in kindergartens, …  (which)  …  is very interesting, because a lot happens in the first years of life.” (P5, physiotherapist)) and that most demonstrate potential for motor learning with the appropriate support measures (“…  I can observe that, overall, a development (learning) is possible for all children  …. But  …  the progress that can be seen is, of course, quite different. So, if I have a child with Down syndrome, that's something completely different  …  than if I have a child who has severe multiple disabilities and has to use a wheelchair.” (P15, physiotherapist). The capacity of children with ID to progress or demonstrate potential for motor learning has been documented by teachers in a study conducted by McDermott, Brick, Shannon, Fitzpatrick, and Taggart [(34); see also (35)].

Concerning psychosocial and cognitive impairments, teachers have observed that students with ID exhibit impairments related to personality (reduced self-esteem), motivational and volitional processes (low motivation, lack of will to get things done), and emotional aspects (anxiety, suffering from the impairment). Relatedly, a few teachers explained: “…  our students are often very reserved and may not dare to try certain things because they have not collected any experience  …  outside of school due to their physical conditions.” (P1, SET), “…  they (students) lack of intrinsic motivation to move and therefore do not  …  start moving on their own.” (P9, physiotherapist), “it (the willingness) is not for a long period  …  they try for a while. If it doesn't work, they quit  …” (P6, SET), “They need a lot of support, …  feel very insecure, …” (P10, SET). Furthermore, students occasionally exhibit unconventional behaviors, including an urge to move, self-harm, aggression toward others, and goal-oriented behavior. One teacher (P1, SET) stated that students with ID and ASD often encounter challenges in group activities: “People diagnosed with ASD generally have difficulty with group games or games with rules because these people tend to be very self-centered and have difficulty understanding and implementing shared rules or group tasks.”. This was described by individuals of this population, youth with ASD and mild ID, interviewed in a study conducted by Boucher, McIntyre, and Iarocci (36).

It is hypothesized that the cognitive impairments observed in students with ID could be attributed to impairments in mental representation and cognitive control processes or executive functions. The following quotes may illustrate this: “I think the body schema is also an issue that is not as present among the students as it is among other students.” (P1, SET), “…  where one would otherwise assume that these movements are automated, they are not automated in our students, or not to the extent that they would be in others of that age  …” (P1, SET)), “…  concentration (is) also (limited) in many things.” (P14, SET), “…  I think it's also difficult for some people to understand the task, to implement what they're given as a verbal instruction.” (P8, SET with a focus on intellectual development). In particular, students with both ID and ASD appear to exhibit difficulties in planning and executing actions, as reported by a SET (P1): “…  for many of them (individuals with ASD), it is simply not possible to understand the movement task and then to implement it into an action. … in many students with autism, action planning … is not yet as developed or as present as in other students …”. These findings are consistent with the results of a study conducted by Panerai, Tasca, Ferri, Genitori D'Arrigo and Elia (37), which demonstrated that children with ASD and ID exhibit deficiencies in action planning.

In summary, the findings on physical, motor, psycho-social, and cognitive impairments in students with ID align with the descriptions provided by the American Association on Intellectual and Developmental disabilities (1) or the APA (2). Moreover, these findings align with the physical and psychological factors that teachers in focus groups identified as influencing physical activity in adolescents with ID (34).

Teachers' perceptions of general characteristics of students emphasize the importance of adapting both motor assessment and interventions to the children and adolescents with ID's individual characteristics and needs, ensuring that they can participate and perform.

Unlike previous studies, this study revealed not only physical-motor characteristics of children and adolescents with ID, but also their psycho-social and cognitive characteristics, based on teachers' perceptions. Therefore, this study expands the understanding of movement behavior of students with ID by emphasizing physical-motor, psycho-social, and cognitive challenges and resources.

### 3.2 Motor skills mastered independently, performed with difficulty, and were wished to be learned by students

#### 3.2.1 Motor skills mastered independently

The teachers indicated that students with ID can perform motor skills independently, such as “washing hands” and “transporting” and handling various objects (toys, balls). Furthermore, students with ID can perform activities such as “running”, moving on playground equipment such as trampolines and slides, “rolling a ball”, and “moving in the water” independently and successfully. These are gross and fine motor skills (38) required in everyday life and for simple sporting activities. Since these activities can be assumed to have largely stationary or predictable environmental characteristics, these motor skills were considered to be performed in a static or predictable environment.

In addition, the teachers indicated that students with ID could engage in activities such as “ball”, “catching”, “running”, and “movement” games and activities involving wheeled/mobile equipment where balance is not a primary requirement. These activities include moving on a “roller board” or using three- or four-wheeled vehicles “tricycle”). These activities were deemed to be performed in a dynamic or unpredictable environment, given that it can be assumed that the relevant environmental context features are likely to be in motion during these activities. The findings align with the descriptions presented by Schott (39), among others, who have reported that children with DS can master general motor skills in everyday life.

Notably, the students have demonstrated proficiency in physical activities conducted in a dynamic and unpredictable environment using mobile sports equipment. This encompasses physical activities with vehicles that possess two, three, or four wheels or rollers and are propelled by cycling or pushing movements with both legs or one leg in either a seated or standing body position (bicycle, “walking bike”, “scooter”, “trike”, etc.). One potential explanation for this phenomenon is the specific type of activity in question. The perception of external forces acting on the body when cornering, accelerating, and decelerating is an inherent aspect of locomotion with moving equipment. This rapid mode of transportation can be an enjoyable experience for students (40). Moreover, it can foster in children the belief that they can engage in or compete with their peers despite any impairments and that they are sometimes as proficient as those who typically outperform them. These factors have the potential to pique the interest of children with ID or motivate them to engage in regular practice, ultimately leading to mastery of the activity.

The results indicate that engaging and encouraging activities that stimulate interest and motivation may be beneficial in promoting movement or physical activity (36) and health (41).

#### 3.2.2 Motor skills performed with difficulty

The teachers' perceptions also revealed that students with ID cannot perform specific motor skills or only do so with assistance. The students with ID were reported to encounter difficulties when eating with cutlery frequently, “tying shoe laces”, “putting on a jacket”, “opening bottle caps”, and walking independently over uneven surfaces (“…  for many (students), …  walking across a meadow is more difficult than simply walking on a path …” (P9, physiotherapist)). Additionally, they were perceived to have motor difficulties when asked to “throw”, “kick”, “hit a ball”, “hop”, or jump over a stationary rope (“…  for example, running and jumping over a rope stretched above the ground while running ….  There are many children who can run, but when they have to jump over something while running, their leg coordination is not sufficiently developed, and they stumble …” (P5, physiotherapist)). This finding can be supported by the results of Simons, Daly, Theodorou, Caron, Simons, and Andoniadou (8) and Schott, Holfelder, and Mousouli (21), investigating TGMD-2 performances in children with and without ID. In these studies, children with mild ID showed poor performance in object control skills, such as throwing, hitting, and kicking, and locomotor skills, such as hopping, compared to TD children. Given that the relevant environmental context features in the reported activities were assumed to be relatively stationary, these motor skills can be considered as occurring in a relatively stable or predictable environment.

In addition, teachers indicated that students with ID face difficulties when attempting to control a moving object or move an object to a moving target, as may be the case when “kicking”, “throwing”, “hitting”, “catching”, or “bouncing”. They also encounter difficulties engaging in games requiring more complex motor skills, such as “ball games” (e.g., soccer, basketball) and racquet games (e.g., “badminton”, “hockey”). Activities involving moving sports equipment that require good balance skills, including riding a bicycle (“…  (riding) a bicycle is simply too difficult for the majority (students).” (P6, SET)) or a stepper (“Pedalo”), are also challenging for this population. These impairments in both gross and fine motor skills are in line with studies by Hartman, Houwen, Scherder, and Visscher (16), Rintala and Loovis (17), Simons, Daly, Theodorou, Caron, Simons, and Andoniadou (8), Westendorp, Houwen, Hartmann, and Visscher (9), and Zikl, Holoubková, Karásková, and Veseliková (18), who examined motor performance in children and adolescents with ID using standardized and norm-referenced motor tests. In these activities, it can be assumed that the environmental context features, such as people, objects, or the ground, are in motion. This is why these motor skills are considered to be performed in a dynamic or unpredictable environment.

The findings underline the importance of including some of the reported movement tasks within assessment tools and developing motor interventions that address the challenges mentioned.

#### 3.2.3 Motor skills wished to be learned

According to educators, students with ID demonstrate a strong desire to acquire proficiency in various daily living activities, including personal care (e.g., “toileting”, “eating”, “dressing”, and “mobility”), food preparation, and household management (“clearing the table”, “filling the dishwasher”). The student's fundamental aspiration is to function independently and autonomously in their quotidian school life, which prompts an endeavor to master fundamental everyday motor skills. In the sports domain, students desire to gain experience in movement in water, in movement landscapes, and with mobile sports equipment such as “roller boards”, “city scooters”, two-, three-, or four-wheeled vehicles, “inline skates”, skateboards, etc. Moreover, they desire to participate in various sporting activities, ranging from more structured games, such as small games, to physically demanding activities like “soccer”.

The findings suggest that using sports equipment and learning about such activities may be beneficial in promoting movement behavior in children and adolescents with ID. This assertion is further substantiated by Davison, Werder, and Lawson's (42) findings, which revealed that children who walk or cycle to school exhibited higher overall daily physical activity levels.

However, the findings of this study on students' physical activity goals or desires are based on teachers' perceptions, which raises the question of whether teachers can accurately determine students' desires or goals. To avoid this problem in future studies, asking students, children, and adolescents with ID directly about their wishes and goals would be advisable. It is important to note that not all children and young people with disabilities can speak (“…  we also have a large student body that cannot express itself verbally …” (P5, physiotherapist)) or articulate their goals.

A closer examination of the data reveals several discrepancies in teachers' perceptions of movement behavior and students' wishes. First, teachers' perceptions of students' abilities to perform specific motor skills and the motor skills students desire are not always aligned. This discrepancy can be attributed to internal factors, such as perceptions, personal norms, or values, which can be influenced by individual movement experiences, and external factors, such as work experiences and the educational environment (43). Second, regardless of the professional background of the teachers, some teachers faced difficulties generalizing their statements, while others did not consider this aspect. Third, while teachers with a pedagogical background tended to describe movement behavior of students from a psycho-pedagogical perspective, thus bringing pedagogical and psycho-social elements to the fore, teachers with a therapeutic background generally elucidated movement behavior from a therapeutic or medical-oriented perspective, highlighting physical and motor factors. These differences between teachers represent the different perspectives that were adopted, through which the movement behavior of students was perceived. Overall, teachers with diverse backgrounds and professional foci generated valuable insights in these topics that complemented each other and aligned with the authors' original intention.

In contrast to earlier research, this study identified motor skills students master, those they face challenges with, and those they wish to learn. Thus, this study contributes to promoting the movement behavior of children and adolescents with ID's movement behavior by highlighting potential skills that could be considered in interventions to reinforce strengths, compensate for weaknesses, and meet desires.

### 3.3 Problems related to movement behavior in students with ID

Furthermore, the present study revealed that children and adolescents with ID may experience individual, environmental, and task-related challenges related to their movement behavior. Teachers believed that some students with ID experience motor difficulties due to physical impairments (“…  the problem in (students with) Down syndrome is often that they are simply too hypotonic to be able to perform movements …” (P6, SET), “For those, it's obviously extremely difficult to grasp a glass, or a ball, or whatever due to their visual difficulties.” (P2, SET with a focus on physical and motor development)) and perceptual problems (“…  they can't pay attention to the lines on the floor because they simply don't perceive them and therefore …  can't participate in something like that.” (P6, SET)), in addition to deficiencies in the development of movement coordination and physical strength (“…  holding a badminton racket and hitting a small shuttlecock fails due to poor postural muscle tone, because you need fairly a strong tone  …  in the back muscles. … Hitting the ball requires eye-hand coordination …  many of our students have a very poorly developed eye-hand coordination, meaning that they  …  don't hit the ball …. Then  …  applying the right amount of force to hit the ball doesn't work because the force regulation is disturbed …  shuttlecock or badminton … is generally not realizable …” (P5, physiotherapist), “But we also have many students who are not even able to hold their own body weight …  and develop sufficient arm strength.” (P12, SET)). These observations were further corroborated by the teachers' assumptions concerning the role of “fear of a ball” (P10, SET), “fear of heights” (P10, SET), and lack of “self-confidence” (P12, SET). Additionally, they noted challenges in understanding, planning, and executing movement tasks (“…  if I can't understand what I have to do …, then I can't physically execute it …” (P6, SET)) and limitations in “attention” span (34). Environmental problems related to movement behavior manifest in students with ID in static and dynamic environments. These observations underscore the complexity of movement behaviors in students with ID, highlighting the need for a multifaceted approach to understanding and addressing these challenges. For instance, difficulties emerge when motor activities are performed in environments where the relevant environmental features (e.g., people, objects, or the ground) are in motion (“… always remember that ‘moving object’ (ball, person) to ‘moving object’ (ball, person) does not work.” (P5, physiotherapist)) and when students with disabilities traverse uneven terrain (“As soon as it becomes uneven and the ground changes, e.g., grass or stones or something like that, they often become very unsure.” (P4, educator)) or are constrained in their mobility by the assistive devices they utilize (“Students who need support while walking or who also have orthoses are more impaired when running …” (P1, SET)). A study conducted by Vuijk and colleagues (24) revealed that children with ID exhibited deficiencies in motor skills, particularly in stationary (e.g., drawing) and dynamic (e.g., catching, balancing) contexts. The investigation further suggests that these difficulties may be associated with the specific task being performed, particularly when students with ID attempt complex movements or tasks that demand high precision (“Most (students) can … use scissors. But they can't use the scissors in a way that would allow them to achieve their goal. And it's similar with a pen or a paintbrush.” (P9, physiotherapist)) or speed (“… walking rapidly is difficult for many students.” (P16, physiotherapist)).

In light of these findings, it is recommended that practitioners prioritize enhancing fine and gross motor skills in both static and dynamic environments for children and adolescents with ID, focusing on addressing movement-related challenges that have been reported and suspected. Particular emphasis should be placed on acquiring and promoting fundamental motor skills to foster independence and enhance participation in social or physical activities (44). Unlike previous research that mainly focused on physical-motor limitations, these findings show that, in addition to physical-motor problems, teachers assumed and mentioned psychosocial and cognitive factors as further individual problems and task-specific and environmental factors as task- and environmental problems related to movement behavior. Thus, this study provides added value by expanding the understanding of problems related to movement behavior to include not only physical-motor factors as individual problems, but also further individual problems (psycho-social) and environmental and task-related problems.

### 3.4 Factors influencing the movement behavior and movement wishes in students with ID

**Figure 1:**
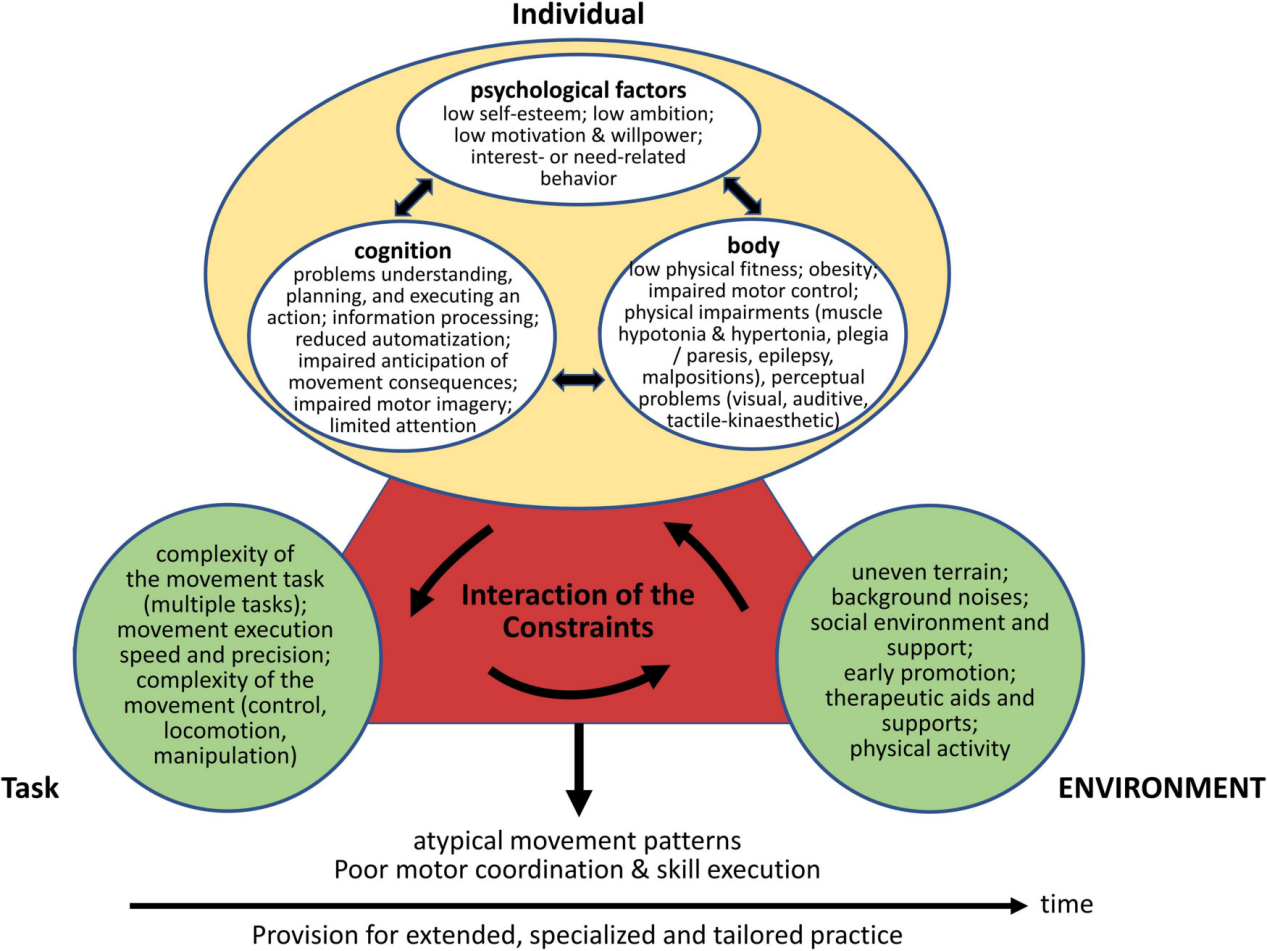
Overview of possible individual, task-related, and environmental factors influencing movement behavior (motor development, motor learning, motor control) in children and adolescents with ID, as assumed by teachers. While the content of the figure refers to the findings obtained within the interview study, the presentation (structure) of these findings is based on the multi-component constraint model introduced by Newell (25) and the model of motor development in individuals with developmental coordination disorder created by Wilson, Smits-Engelsman, Caevenberghs, and Steenbergen (45). The time axis shown in this figure aims to illustrate changes in movement behavior, influenced by the interaction of individual, task-related, and environmental constraints. The concluding banner (“Provision for extended, specialized and tailored practice”) represents a practice-related implication derived from the synthesis of findings, rather than an empirically coded outcome.

Teachers' perceptions indicate that several individual, task-related, and environmental factors influence the movement behavior of students with disabilities. Figure 1 illustrates these factors believed to influence movement behavior and the general characteristics of students with ID reported. The presentation of the results is based on the constraint model for the development of movement coordination developed by Newell (25), which contains core components from dynamical systems theory. Figure 1 was adapted from Wilson, Smits-Engelsman, Caevenberghs, and Steenbergen (45), who created a multicomponent constraint model of motor development in individuals with developmental coordination disorder. This framework was applied to children with ID, as the illustrated mechanisms and challenges are conceptually comparable. Moreover, this framework was applied to emphasize parallels (similarities) while considering population-specific differences. The results presented in this figure may provide a basis for describing, explaining, manipulating, and predicting movement behavior in children and adolescents with ID. Given the potential for movement behavior in students with ID to be influenced by a range of individual, task-specific, and environmental factors, it is imperative to consider the task, environmental context, and individual characteristics of the person when describing, explaining, manipulating, and predicting movement behavior in children and adolescents with ID. In qualitative descriptions of movement behavior, e.g., in school reports, it is important to consider the required movement behavior and the actual movement behavior demonstrated by the performer. In addition to these two factors, it is crucial to consider the characteristics of the performer and the environmental context in which the task is performed. In this way, the actual psychomotor behavior of the performer can be precisely mapped and made comprehensible to third parties. For explanatory purposes, it is crucial to consider the interaction of the core components that influence movement behavior. An explanatory approach to movement behavior based on a multifactorial perspective has the advantage of considering the individual as a whole and explaining an individual's movement behavior (motor development, learning, and control) based on the interaction of changes or factors at different levels.

Physical-motor goals or desires of students with ID are theorized to be influenced by many individual, task-related, and environmental factors analogous to those that shape movement behavior. Individual factors that teachers identified as potentially influencing physical-motor goals or desires were classified as physical, motor, psychological, and cognitive factors. The physical-motor factors encompassed the level of physical and motor development (“…  it depends on how fit they are.” (P14, physiotherapist)) and the “extent of (physical) impairment” (P15, physiotherapist). Psychological factors included a sense of belonging (“Something like belonging, …  if I can play football, then I'm more likely to play in the schoolyard, and then I'm more likely to be part of the team …  or the sports club. So, social belonging or belonging to the … peer group is …, I think, …  an incentive for students.” (P1, SET)), independence or self-determination, the urge to discover, the need for recognition (“I prove myself to others, score goals. This has a very high incentive to get recognition.” (P12, SET)), and career aspirations. In addition to these factors, it was hypothesized that cognitive factors, such as cognitive development (“The degree of disability …  the intellectual disability, plays a role …  in order to develop goals for yourself, …  independent goals, … you have to be able to understand to some extent what it's about …” (P15, physiotherapist)) and perception (“… the children (students) … enjoyed learning to ride a bike …. Of course, this also depends … on what degree of a perception disorder they have. … If you have a perception disorder affecting the spatial area, it's naturally difficult if you can never estimate how high this is or even the speed … (P15, physiotherapist)), may also influence students’ physical goals or aspirations with ID. Regarding the environmental factors, teachers suspected that the social environment (“those people that are around (the students) … the staff at school, and to a large extent, the parents.” (P3, physiotherapist)) and social support, physical activity events/courses, school equipment, and the general availability of materials may influence the students' goals and aspirations.

Furthermore, factors associated with the physical activity task, including the perceived usefulness, appeal (“… if the movement (task) is boring, then I don't do it. If I enjoy it, then I'm … much more interested in continuing to do it. … You also notice that … when they do certain tasks, be it math exercises. As soon as you introduce a form of movement they enjoy, they're much more involved than sitting at a table and having to fill out something silently. (P6, SET)), and efficacy of the physical activity task, as well as the structure and format of the physical activity measures (“In soccer, it's the competitive spirit. I prove myself against others, score goals.” (P12, SET)) (competitive spirit), have been shown to influence students' motivation, objectives, and aspirations. These findings align with the observations of McDermott, Brick, Shannon, Fitzpatrick, and Taggart (34), who reported that team activities and competitions may motivate students with ID.

Considering the extant literature on the factors influencing movement behavior and physical activity aspirations of students with ID, it can be posited that a subset of the aforementioned factors may be employed to promote movement behavior or stimulate interest in physical activity among children and adolescents. As indicated by teachers, the most salient factors influencing movement behavior and the desires of students with disabilities are the social environment and social support. This finding is of particular significance for parents, family members, and teachers, who comprise the primary social environment of children and adolescents. Consequently, their behavior can play a pivotal role in promoting physical activity and stimulating interest in physical activity (46). Parents and teachers can promote the movement behavior and motivation of children and adolescents with intellectual disabilities by providing them with various opportunities for physical activity and encouraging their interests (47) in the context of physical education. Parents and educators can also play a central role in promoting independence, facilitating peer interactions, and providing comprehensive support from an early age. To optimize support for students with ID, there should be regular communication between parents and teachers (48, 49), with teachers having an advisory role in education matters.

Furthermore, individuals responsible for overseeing clubs or commercial sports providers are encouraged to develop exercise opportunities for individuals with disabilities of all ages. This initiative will contribute to the promotion of these individuals, as there is currently a dearth of such opportunities in clubs and commercial sports providers for individuals with disabilities compared to individuals without disabilities. Public institutions entrusted with promoting health and physical activity are also regarded as instrumental in developing sports facilities and creating publicly accessible exercise opportunities. These institutions are considered a crucial source of financial support for schools and parents, thereby underscoring their pivotal role in promoting health and well-being.

While earlier research concentrated on isolated aspects, this study emphasizes the interaction of a range of individual, task-related, and environmental factors influencing the movement behavior of students. Thus, this study contributes to a broader understanding by indicating how these interrelated factors influence students' movement behavior.

### 3.5 Content related to physical-motor and therapeutic activities in schools for students with ID

Regarding the content or motor skills incorporated by teachers in their lessons, teachers reported that various sports and movement activities and different forms of therapy are offered as part of the physical education curriculum in schools for students with ID. Sports and physical activities offered at schools include various activities performed in regular physical education classes, interest-and need-specific activities (running group, wheelchair sports, sports for advanced, etc.) conducted in separate courses, and activities performed in the context of cooperations between schools and clubs (sports clubs, circus). Forms of therapy offered in everyday school life, e.g., in classrooms, the school building, the schoolyard, or in separate therapy sessions in therapy rooms, include physiotherapy, occupational therapy, speech therapy, and riding therapy.

The teachers indicated that students generally have the opportunity to engage in a wide range of physical and movement experiences in physical education classes, including, but not limited to, learning or practicing FMS, motor skills with movable sports equipment (e.g., rolling, riding, and gliding equipment), motor skills with gymnastics equipment, motor skills in water, and sports games. They highlighted the significance of motor coordination training, encompassing a range of skills including “walking”, “running”, “jumping”, and maneuvering with movable sports equipment (e.g., bicycles, Kettcars, skateboards) in addition to object control skills such as “rolling (a ball)”, “bouncing (a ball)”, and handling objects. The therapeutic measures employed in educational settings include respiratory therapy, gait training, mobility training, vibration training, the provision of assistive devices, massage therapy, and the promotion of basic and instrumental activities of daily living. The “Bobath” concept, the “Feldenkrais method”, the “Castillo-Morales” concept, and “Shiatsu” are frequently utilized therapy concepts in practice.

The teachers expressed that physical activity is not only considered in physical education classes during everyday school life, but also in other classes conducted in the classroom or during recess in the schoolyard. Moreover, they stated that movement landscapes or environments are often used to promote the movement behavior of students.

The findings on content related to physical and therapeutic activities suggest that specific physical activities that occur in school contexts, in which students participate, should be considered within motor assessments. This may ensure that children with ID can participate and perform, as the demands of such movement tasks are adapted to the children's abilities and skills. Moreover, interventions should be tailored to enhance students' participation and autonomy in school contexts.

This study revealed which physical activities promote students' movement behavior and how teachers adapt physical activities to meet students' individual characteristics, needs, and preferences. Hence, this study advances knowledge by showing which activities may be considered when designing tailored motor assessments and interventions.

### 3.6 Teaching approaches within sports, movement, and therapeutic activities in schools for students with ID

In the context of pedagogical practices for students with disabilities, teachers indicated that the focus of physical activities or therapeutic interventions should be to promote the enjoyment of movement and support student independence (50), as shown in the following quotes: “… my primary goal is … that the child enjoys physical activity …” (P15, physiotherapist), “… you simply have to focus on having fun, not on training success, …. That's the most important thing because otherwise they don't move a lot. And if you can get them to enjoy moving and have a good time in physical education, … then you've already achieved something. And if you can … get them to do that now and then in their free time, or maybe do a bit of inline skating or cycling, then you've achieved even more.” (P10, SET), and “… the ultimate goal … is independence … so that … he (student) will be able to … live an independent life as good as possible, … even if this only means being able to undress … or dress himself.” (P15, physiotherapist). In this regard, Hall (51) underscores the significance of fostering autonomy in children with disabilities within the classroom environment.

Additionally, the importance of articulating the relevance of a movement task or establishing a connection between a movement task and daily life as a motivating factor for students was underscored, as stated by a physiotherapist (P16): “… some kind of agreement must be made so that … the student sees the purpose of this (movement task) or why he should do it, sometimes even with the parents …”. Teachers expressed the importance of adopting an individualized (“… it's important to simply consider different goals for each student and, … to tailor the activity to each student. I think that's the key for everything, whether it's physical education or movement in the classroom …” (P8, SET with a focus on intellectual development)) and gradual methodological (“… gradually increase …” (P10, SET)) approach to movement learning tailored to students' distinct needs or characteristics (“… to consider the individual characteristics of the student” (P3, SET)). Relatedly, one teacher (P5, physiotherapist) added and put it into a nutshell: “All the movement steps that we can teach a non-physically or a non-mentally disabled person in a single movement sequence, we have to divide into very … small steps for people with mental and physical disabilities. Always maintain an external focus; always keep in mind that “moving object” to “moving object” doesn't work. So, it's best to practice from a standing position, then while walking, then while running …. These (aspects) are absolutely important—(to consider) very … small steps, and then putting the movements together.” Another teacher (P1, SET) echoed this general method, arguing: “In my opinion, I have to reduce the lesson … (demand) in a way or consider small steps that every student can have movement experiences.” Furthermore, the teachers stated that the movement tasks should be systematically adapted to the situational conditions or environmental context (“… to design and modify the activity accordingly.” (P8, SET with a focus on intellectual development)) and the individual needs of the students (“… this requires rather simple forms of play that bring quick fun or are funny …” (P10, SET)). The findings on an individualized and gradual methodological approach to motor learning are consistent with the conclusions of McDermott, Brick, Shannon, Fitzpatrick, and Taggart (34), based on the results of focus groups with teachers, and with the descriptions of adaptive physical education by Vickerman (52) and Winnick and Porretta (53).

In general, teachers consider it essential to promote the movement behavior of students with ID by implementing goal-oriented (“Actually, I think pretty much everything has to be connected to a purpose.” (P10, SET)), functional, relevant (“The relevance (of the motor skill) in everyday life is important.” (P15, physiotherapist)), attractive, interest-oriented, effective (“… they should enjoy doing it (the movement task) and, of course, it should be effective for them.” (P14, physiotherapist)), and promising movement tasks (“feeling of success”). They recommend supporting the learning process through brief instructions (“… not to talk too much! Yes, that's a big mistake that many teachers and adults make: they think they must explain, analyze, and speak a lot.” (P2, SET with a focus on physical and motor development)), demonstrations (“From a methodological point of view, it is important for our students to visualize the task … by demonstrating it.” (P12, SET)), feedback (“If I feel like I'm not getting anywhere, I make a video recording. I show this to the students, … and you will be amazed at how well they recognize their behavior, body language, and motor problems, and how well they can implement it afterward.” (P2, SET focusing on physical and motor development), and reflections (“Learning from the model is a big topic, at least in my lessons. I show the students what I want, and they imitate it. Then, we find out what the problems are and what the difficulty is.” (P2, SET focusing on physical and motor development). Furthermore, they stated that teaching and learning aids (““Jump into the water from the pool's edge” don't work. You take … a large ring, put it in the water, and say: “Jump into the ring.” Then the student can carry out the movement task—external focus.” (P5, physiotherapist), visualization tools (“… what is important is an illustration, … it should not be a purely theoretical teaching. But rather, with the help of symbols and pictures, make it clear to the children what is actually required.” (P13, SET)), therapeutic aids (“… whether it's an orthosis or whether it's a rollator … or an NF-walker or … that you think carefully about what you can include so that a motor progress is actually possible …” (P7, SET with a focus on physical and motor development)), and general assistance or support (“…  I think patience is the basic theme with our students, that you show something maybe 3000 times and show it tirelessly until … this movement can perhaps be carried out independently at some point … and then … accompaniment, hand guidance, training together, execution of the movement together …” (P9, physiotherapist)) in motor learning can be beneficial. As previously mentioned, the motivation to engage in physical activity is enhanced when the activities are enjoyable (34). Furthermore, it has been proposed that encouraging participation in enjoyable physical activities can facilitate the adoption of a more active lifestyle (54). Supportive measures or aids can also enable the motor learning process (53).

One teacher (P6, SET) emphasized the significance of promoting movement behaviors in students with ID through an interdisciplinary approach, given the heterogeneity of diagnoses, symptoms, and needs among this population (52): “I think there should be … a group that discusses this together and … develops ideas.” (P6, SET). Another teacher (P12, SET) underlined the importance of the collaboration between teachers within an interdisciplinary team to promote the movement behavior of students, illustrated by the following quote: “… of course, as a teacher, there's always contact with the physiotherapists, from whom you get a lot of information …. And physical promotion is … a big part of the … classroom work …”. One (P16, physiotherapist) of several teachers also emphasized that students require sufficient time to practice to make progress: “… it often takes a lot of repetitions to … achieve success.” (P16, physiotherapist), corresponding to the findings of McDermott, Brick, Shannon, Fitzpatrick, and Taggart (34) based on teacher reports. Given the considerable heterogeneity among students, the teachers underscored the value of offering group-based movement classes in the school context, focusing on each student's specific motor performance characteristics. This approach is believed to provide optimal support for students with diverse needs, which the following quotes may illustrate: “… I think it's really good to simply divide the students into … groups, like the swimming group, … or …  advanced sports or wheelchair sports because I think that's something where you can work in a really goal-oriented way.” (P8, SET with a focus on intellectual development), “I think what's important, since our student body is so heterogeneous, is that you form groups … one high-performing group, one group … with the lower-performing students, because otherwise the high-performing students are quickly held back by the weaker ones if the activity is open to everyone.” (P10, SET). Teachers generally believe that students with ID benefit from physical activity in different ways (34). Therefore, in addition to physical education, it may be beneficial to incorporate physical activity into regular classes and recess (55).

In general, the teachers considered it important regarding the organization of physical education classes that teachers actively participate in the lessons themselves and act as movement role models and motivators (“Teachers serve as movement role models and motivators. When teachers actively participate in physical education themselves, it supports the students’ willingness to perform.” (P12, SET); “ … you have to be able to involve them somehow. You have to be a little emotional too. You have to cheer them on … as a teacher … You also have to show them that it's … cool … to move …” (P10, SET)). Furthermore, it was recommended that the lead teacher is responsible for the instruction, while the other teachers present during physical education classes are primarily responsible for supervising the students (“Generally speaking, I would say that the person who is responsible for the lesson has the task of providing the overall framework, and those who are there with the students have to assess what support each student requires … then the person who is responsible for the lesson also has the opportunity to give tips on what can be improved …, how things might be made easier …” (P1, SET); “… many students simply need direct support …” (P6, SET)). Moreover, the teachers emphasized that it is important for many students to consider a clear and recurring lesson structure and to present the structure of the lesson unit at the beginning, as illustrated by the following quotes: “…  I always think it's quite good when it (the lesson) has a relatively structured schedule, because our students need this, and they can then engage with it better when it's more ritualized. Then they know what to expect there, what's coming, and (they) can then participate better and better and adapt to it.” (S6, SET); “…  an introduction, a main part, a closing part, … simply structured.” (P8, SET); “The structure … You present it (the structure of the lesson). I think it's  … very good that it's … clear what's coming when.” (P4, educator).

The teachers' perceptions of teaching approaches in schools for children with ID highlight the need for an individualized and gradual methodological approach to promote movement behavior of students with ID using attractive and promising movement tasks.

In light of the previous findings (code e and f), the present study has shown that physical education in schools for students with ID is based on a multi-layered and interdisciplinary framework that includes a wide range of sport, exercise, and therapy modalities to meet the specific needs of these students. In this context, it can be assumed that the national education plan or the school curriculum and the requirements of the responsible state educational institutions primarily determine the overarching concept of physical education and the specific content.

Furthermore, regarding the discrepancy in teachers' perceptions related to the content taught by teachers and the content desired by students, the study has revealed that the students' expressed physical activity wishes are primarily reflected in the curriculum or considered by teachers. Relatedly, it can be assumed that supporting students' interests in the development process is conducive to their personal development and fulfills their wishes.

In contrast to previous research focusing on other pedagogical aspects, the findings of this study underline the importance of using attractive, promising, interdisciplinary, and individualized approaches to increase motivation and enjoyment of movement, to reduce the demands of movement tasks, and overall to promote movement behavior. Thus, this study expands understanding by demonstrating a need to consider promising tailored approaches to promote movement behavior.

The results of the study suggest that the movement behavior (motor development, motor learning, motor control) of children with ID results from the interaction of individual, task-related, and environmental constraints or factors. Understanding the influence of the interaction of these factors is crucial for promoting movement behavior. While factors, such as overly complex tasks or inappropriate environmental influences, for example, presumably negatively influence person-related processes (motivation, understanding, concentration) and thus movement behavior, factors, such as supportive instructional measures, aids, and support measures, for example, may reduce task demands and therefore presumably promote person-related processes (motivation, understanding, concentration) and movement behavior.

The interrelated mechanisms highlight that movement behavior is influenced by individual, task-related, and environmental constraints, rather than individual factors alone. This suggests that individual, task-related, and environmental factors should be considered when describing, explaining, manipulating, and predicting the movement behavior of an individual. It also emphasizes the importance of using a systematic multi-disciplinary approach that intends to specifically manipulate modifiable factors influencing movement behavior to promote movement behavior of children and adolescents with ID effectively and efficiently.

Although the study's findings are based on teachers who worked in special school settings, the insights provided are also relevant for inclusive mainstream contexts and can be transferred to these contexts. Factors or practices, such as an individual and gradual learning approach, visualizing means (demonstration, photos, videos), and supportive measures (aids and support), may support motivation, participation, and independence in these contexts. Furthermore, using a systematic multi-disciplinary approach to promote movement behavior is equally crucial in such contexts.

Despite the strengths of this study, some limitations need to be mentioned and acknowledged in interpreting the findings. One limitation concerns the sample drawn exclusively from a German school context. This may be associated with limitations of the transferability of the findings to educational systems in other countries or other cultural settings. Moreover, the recruitment of the participants was directly carried out by the researcher, who selected the individuals based on the presumed ability to address the research questions and to contribute to relevant insights. This recruitment strategy may have introduced the possibility of self-selection bias, as those who participated could differ systematically from those who did not regarding factors such as expertise, experience, fluency, or openness. A further limitation relates to the inclusion of teachers with diverse backgrounds. While including teachers with diverse backgrounds and expertise may have enriched the dataset, it may have biased the difference between group-specific perspectives. The findings should also be interpreted cautiously, as data analysis was carried out primarily by a single researcher, which may induce interpretative bias, despite efforts to ensure rigor and reflexivity. Relatedly, it needs to be noted that qualitative analysis inherently involves interpretative work and is influenced by the researchers' perspectives. Thus, the findings obtained within qualitative analysis are automatically shaped by the authors' knowledge and experiences, even when rigor is very important in the evaluation process. Finally, the study lacks methodological or observational triangulation, limiting the possibility of corroborating the results through multiple data sources or approaches. Despite the limitations mentioned above, this study provides valuable insights into the movement behavior of children and adolescents with ID and represents a solid foundation for developing motor assessments and interventions. Future research could build on the findings obtained by considering more diverse samples, multiple analysts, and triangulated methods. This would help improve the research quality and broaden the understanding of movement behavior.

## 4 Conclusion and future directions

This study explored teachers' perceptions of students with ID from a movement perspective to understand how to develop appropriate motor assessment instruments and motor interventions to promote movement behavior in children and adolescents with ID. To examine teachers' perceptions, qualitative interviews were used to interview teachers from several special schools using a qualitative study approach. A dual conceptual framework combining Newell's constraints model (25) and Gentile's taxonomy of motor skills (26) was employed, which guided the analysis. The study explored six central research questions to address the study's aim.

Regarding RQ 1, the findings indicate that students show different levels of motor development and that most students show potential for motor learning. According to the teachers' perceptions, students can generally be considered a remarkably heterogeneous population in terms of their individual characteristics (cognitive, psycho-social, and physical-motor characteristics).

Concerning RQ 2, the data revealed that students are generally able to perform simple everyday activities and are keen to learn or perform activities that help them become more independent and self-determined. Teachers' perceptions also indicated that students struggle to perform fine and gross motor activities in static and dynamic environments.

Concerning RQ 3, the analysis demonstrated that students' problems related to movement behavior may be associated with individual, environmental, and task-related factors.

Addressing RQ 4, the findings suggest that students' movement behavior and desires are influenced by a range of individual, task-related, and environmental factors, as pointed out by the teachers.

RQ 5 is answered by the findings, indicating that various sports and movement activities, as well as different forms of therapy, are offered at German special schools for students with ID.

Finally, RQ 6 is informed by results demonstrating that it is important to use an individualized and gradual methodological approach to promote students' movement behavior in the context of teaching practices, as underlined by the teachers. In summary, the findings answered the research questions and provided a deep insight into how students with ID can be assessed and supported in practice. These are based on teachers' extensive experience with students with ID, which lends them a comprehensive and meaningful quality. They offer valuable insights that can inform both theoretical and practical applications. Moreover, they are more meaningful than the results of a single motor assessment of a specific number of individuals, as has been the case in many previous studies. This may be due to the extensive number of students as perceived by the interviewed teachers across different school settings and different perspectives in the course of their work.

Given the overall study objective and the findings obtained based on the research questions above, several conclusions can be drawn regarding motor assessments and interventions.

The empirical findings primarily related to RQ 1 and RQ 2 indicate that motor assessments and interventions must be adapted to children and adolescents with ID's specific needs, characteristics, and preferences to facilitate their participation and performance. Using tasks pertinent to the autonomy and involvement in everyday life for children and adolescents with ID, or tasks that enable the monitoring, acquisition, and enhancement of skills beneficial to the development of children and adolescents with ID, is a practical approach to achieve this objective.

In addition, evidence primarily emerging from RQ1, RQ 5 and RQ 6 suggests that tasks perceived as attractive and promising by the individuals in question, which, therefore, arouse their interest and increase their motivation, can be considered appropriate. Moreover, using assistive technology in assessment and intervention may be essential to reduce motor and cognitive demands, increase motivation and self-confidence, and prevent accidents and injuries in children and adolescents with ID.

In general, the findings reported in relation to RQ1, RQ 3 and RQ 4 indicate that it may be crucial to consider both systematic, structured assessments to accurately assess motor functions in children and adolescents with ID and interventions that provide them with a systematic, methodical approach to acquiring motor skills or enhancing motor skill performance. For these purposes, Gentile's taxonomy (26) could be used to differentiate motor skills based on the variability of the environmental context and movement execution.

Finally, insights obtained from RQ 1 and RQ 2 indicate that it may be necessary to consider further criteria, such as the intentional behavior (intentional engagement in activity), the outcome of movement behavior, and the level of independence of an individual, in the context of the assessment of the developmental progress in children and adolescents with ID. Assessments considering these criteria would provide valuable information about an individual's psychomotor profile.

Future research could focus on the development of appropriate instruments to assess the psychomotor abilities of children and adolescents with intellectual disabilities. These instruments could be both subjective and objective and could be used by teachers, therapists, parents, or guardians. In addition, future research could focus on developing theory-based, systematically structured approaches to facilitate motor skill acquisition and improve motor performance. These approaches need to be effective, engaging, and easy to use. They would also need to include cognitive-motor and physical-motor interventions.

## Data Availability

The raw data supporting the conclusions of this article will be made available by the authors, without undue reservation.
