# Supplementary material for: A qualitative exploratory study of teachers’ perceptions of movement behavior of students with intellectual disabilities in the school context
Source: Front Sports Act Living. 2025 Nov 4;7:1655758. doi: 10.3389/fspor.2025.1655758 (PMC12623388; doi:10.3389/fspor.2025.1655758)
Supplement: Supplementary file 1 [file Datasheet1.docx]

Supplementary Material

# 1 Interview guide

**General perceptions / observations**

1. What observations can be made regarding students' movement behavior in the school context, e.g., during classes in the classroom, gym, recess in the school building, or in the schoolyard?

**Motor proficiency / mastery**

2. What are the everyday and athletic movements that students perform with the most significant degree of independence and success?

3. Are there similarities between students in terms of motor skills? If so, what are they?

4. What factors could potentially influence the execution of everyday and athletic movements or motor performance?

**Motor problems**

5. Which fundamental movements or skills, whether for everyday activities or sports, do students typically encounter difficulties in performing?

6. What specific challenges do these movements or skills present for the students? Provide a detailed description of the nature of the problem associated with each movement or skill.

7. Are there any commonalities among the students concerning the difficulties they face? If so, what are they?

**Movement wishes / physical-motor goals**

8. What are students' most popular physical activities, both in terms of everyday movements and athletic pursuits?

9. What factors could have an impact on physical-motor goals or desires?

**Physical education content at schools for students with ID**

10. What physical activities are typically taught in physical or physical-motor education classes?

**Teaching approaches in physical education lessons with students with ID**

11. What are the key considerations when teaching physical education to students with disabilities?

# 2 Code tables

**Table 1.** *Tabular presentation of the thematic main code with subcodes. Numbers in brackets indicate the number of codings.*

| **general characteristics in children and adolescents with ID** | | |
| --- | --- | --- |
| **motor function** | **psyche und social behavior** | **cognition** |
| - motor development - developmental characteristics - missing specific motor milestones (n=1) - motor developmental delays (n=8) - persistent early childhood reflexes (n=2) - stagnation or deterioration of motor performance during puberty (n=4) - group-or diagnosis-related characteristics - heterogeneous population regarding motor skills performance (n=22) - motor behavior within the range of possibilities (n=6) - dependence of therapeutic aids and general support (n=12) - ability-, skill-, or requirement-related characteristics - reduced reaction ability (n=3) - reduced balance ability (n=23) - impaired force control (n=6) - impaired movement coordination (n=66) - reduced physical fitness (n=8) - reduced speed ability (n=2) - reduced endurance ability (n=8) - reduced strength ability (n=9) - reduced flexibility ability (n=1) - difficulties in executing complex movement tasks (multiple tasks) (n=14) - difficulties in executing movement tasks demanding high precision (n=11) - difficulties in executing movement tasks demanding high movement speed (n=2) - difficulties in executing complex movements (control, locomotion, manipulation) (n=10) - motor learning and motor control - significant progress in learning during the early years (n=3) - potential for motor learning with appropriate support (n=4) - difficulties in executing motor actions following verbal instructions (n=7) - difficulties in executing motor actions following visual instructions (n=3) | - psyche - characteristics related to personality - impaired self-perception (n=2) - low insight to perform physical activity (n=4) - low risk awareness (n=1) - low self-esteem (n=7) - openness for help (n=1) - low ambition (n=4) - high sensitivity (noise sensitivity) (n=2) - motivational and volitional aspects - low motivation (n=3) - low willpower (n=4) - emotional aspects - anxiety (fear of the ball, fear of unknown situations, fear of heights) (n=6) - enjoyment of movement (n=1) - suffering from the impairment (n=2) - behaviors / actions - interest- or need-related behavior (n=5) - result- or purpose-oriented behavior (n=3) - excessive eating during puberty (n=1) - effortless behavior (n=7) - low physical activity behavior (n=4) - hyperactivity / urge to move (n=6) - hetero- and self-aggressive behavior (n=2) - social behavior - behavioral problems during group games (n=2) - difficulties in interacting with peers (n=3) - challenges in managing personal space and proximity (n=1) | - cognitive developmental delays / impairments (n=2) - difficulties in understanding the movement task (n=17) - problems in planning and executing actions (n=12) - perceptual disorders or difficulties (cognitive information processing) (n=14) - difficulties in recognizing the affordance or invitation of an object (affordances) (n=1) - impaired action (motor) imagery (n=2) - reduced automatization of movement sequences (n=4) - limited attention (n=4) |
| **body** | | |
| - individuals with additional types of impairments (physical, motor, psycho-social) (n=22) - individuals with additional ICD diagnoses (Down-Syndrom, Epilepsy etc.) (n=88) - increase in physical impairments due to improper leading (overload) and physical inactivity (n=5) - weight gain during puberty (n=2) | | |

**Table 2.** *Tabular presentation of the thematic main code b with subcodes. Numbers in brackets indicate the number of codings.*

| **Every-day and sports-related motor skills that students with ID have mastered, struggle to master, and would like to learn.** | | | | |
| --- | --- | --- | --- | --- |
| **characteristic features of motor skills / activities** | | **motor skills / activities that can be carried out largely independently and successfully by most students** | **motor skills / activities that are not performed independently or performed with difficulty by most students** | **motor skills / activities that most students wish to learn or perform** |
| **movement in a stable and / or predictable environment** | self-care-activities | - washing hands (n=1) - toileting (n=3) - transfers (n=3) - drinking (n=2) - eating (n=5) - dressing and undressing (n=3) | - toileting (n=2) - transfers (n=5) - drinking (moving the glass to the mouth; tilting a glass) (n=2) - eating (moving the spoon / fork to the mouth; scooping food) (n=3) - putting shoes on (n=1) - tying shoes (n=5) - putting on socks (n=2) - opening and closing buttons (n=5) - opening and closing (threading) a zipper (n=6) - putting on and taking off pants (n=3) - putting on and taking off a jacket or pullover (n=4) | - washing hands (n=1) - brushing teeth (n=1) - washing / showering (n=2) - toileting (n=4) - eating (n=6) - dressing and undressing (n=5) - tying shoes (n=2) - putting on a jacket (n=1) |
|  | activities of daily life / household activities | - lying (n=1) - standing (n=1) - sitting (n=3) - standing up (at the table) (n=3) - sitting down (n=1) - moving / locomotion (crawling, creeping, rolling, driving with a wheelchair) (n=8) - walking forward (n=16) - going upstairs and downstairs (n=5) - gesticulating / signing (n=1) - grasping and holding (n=5) - unlocking sth. with a key - setting the table (n=2) | - crawling (n=1) - walking forward (n=1) - walking sideways (n=2) - walking backwards (n=3) - going upstairs and downstairs (n=6) - grasping and holding (n=4) - unscrewing a bottle cap (n=1) - bending over / picking up sth. (n=3) - hanging up a jacket (n=1) - setting up an ironing board (n=1) | - sitting (n=1) - standing (n=1) - moving / locomotion (walking / walking around / driving with a wheelchair) (n=5) - standing up (bed) (n=1) - going upstairs and downstairs (n=1) - holding a plate and walking around with it (n=2) - setting and clearing the table (n=3) - cutting / preparing a piece of bread (n=2) - cooking (n=1) - loading the dishwasher (n=1) - opening and locking a door (n=1) |
|  | motor skills in a classroom | - overcoming obstacles (fixed objects) (n=1) - opening a school bag (n=1) - handling objects (toys, balls) (n=2) - doing a jigsaw (n=1) - rattling (n=1) - writing (n=1) - transporting (n=6) - ironing (n=1) - sanding (n=1) - sawing (n=1) - cleaning (n=1) | - overcoming obstacles (n=4) - forming a ball (n=1) - sawing (handicrafts) (n=1) - doing a jigsaw (n=1) - writing / writing neatly (n=4) - painting (n=1) - accurate clipping (n=2) | - opening and closing a backpack (n=1) - opening a lunch box (n=1) - transporting (n=1) |
|  | exercise in leisure time / physical education | - running (n=11) - trampolining (n=5) - jumping (n=1) - hopping (n=5) - turning in circles (n=1) - sliding (n=2) - boxing (n=1) - climbing (n=4) - swinging a towel (n=1) - rolling a ball (n=2) - moving in water (swimming) (n=4) - throwing (n=9) - kicking / shooting (n=4) | - running (n=2) - long jumping (n=2) - long throwing (n=1) - doing gymnastics (n=4) - moving in a movement landscape (n=2) - jumping off objects (n=1) - climbing (n=3) - running on a treadmill (n=2) - Yoga (n=1) - hopping (n=3) - rope jumping (n=1) - trampolining (n=4) - one-legged stand (n=1) - walking on a line (n=1) - balancing on a bench (n=3) - slipping through sth. without touching (n=1) - swimming (staying afloat / mastering swimming techniques) (n=6) - targeted kicking / shooting (target kicking) / passing (n=2) - targeted throwing (target throwing) (n=3) - kicking (n=1) - throwing (n=9) | - sliding (n=2) - creeping (n=1) - crawling (n=1) - progressing hand over hand (n=1) - running (n=5) - rope jumping (n=1) - trampolining (n=3) - somersault (n=1) - tricks (n=1) - moving in a movement landscape (n=1) - climbing (n=5) - strengthening (n=1) - gymnastics (n=1) - rolling a ball (n=1) - swimming / moving in water (n=6) - throwing (n=1) - kicking / shooting (n=2) |
| **movement in a dynamic and / or unpredictable environment** | activities of daily life |  | - walking on uneven grounds or changing terrain (n=5) | - hiking (n=1) |
|  | exercise in leisure time / physical education | - ball games (n=2) - table tennis (n=1) - table soccer (n=1) - basketball / wheelchair basketball (n=1) - soccer (n=5) - catching games (n=1) - running and movement games (n=1) - swinging (n=7) - dancing (n=1) - agility activities (n=1) - moving with a rolling board (n=2) - moving with a scooter (n=2) - driving with three or four-wheeled vehicles (trike, tricycle, Kettcar, bike) (n=10) - riding a bicycle (n=1) - throwing (interpretation of the authors) (n=5) - catching (n=5) - kicking / shooting (interpretation of the authors) (n=2) | - ball games (n=2) - tennis (n=3) - table tennis (n=1) - shuttlecock / badminton (n=3) - hockey (n=1) - table soccer (n=1) - balancing on a moving band (n=1) - dancing (n=1) - riding a bicycle (n=4) - moving with a stepper (n=2) - targeted kicking / shooting (target shooting) / passing (interpretation of the authors) (n=2) - targeted throwing (target throwing) (interpretation of the authors) (n=2) - kicking / shooting (interpretation of the authors) (n=2) - throwing (interpretation of the authors) (n=4) - bouncing (n=2) - catching (n=11) | - ball games (n=3) - soccer (n=11) - catching games (n=3) - riding (on horses) (n=2) - swinging (n=5) - dancing (n=2) - moving with a rolling board (n=2) - moving with a scooter (n=2) - driving with three or four-wheeled vehicles (trike, tricycle, Kettcar, bike) (n=5) - riding a bicycle (n=8) - skateboarding (n=1) - inline skating (n=3) - throwing (interpretation of the authors) (n=2) - catching (n=1) - kicking / shooting (interpretation of the authors) (n=1) |

**Table 3.** *Tabular presentation of the thematic main code c with subcodes. Numbers in brackets indicate the number of codings.*

| **problems related to movement behavior of students with ID** | | |
| --- | --- | --- |
| **characteristic features of the problems** | | **problem related to movement behavior** |
| **individual problems** | **physical-motor problems** | - impairment-related problems |
|  |  | - problems in executing object control and locomotor skills due to neuromusculoskeletal impairment (n=9)   (problems in climbing stairs due to muscle hypertony or hypotony; walking with much effort due to ataxia, scoliosis, clubfoot, or claw toes; problems in upright standing due to muscle hypotony, etc.) |
|  |  | - problems in executing object control and locomotor skills due to overweight (n=2)   (problems in picking up objects due to excessive belly fat; performing for long periods of time only with great difficulty due to being overweight) |
|  |  | - functional and movement limitation due to atypical changes in joints and muscles (n=5)   (problems performing motor activities, such as getting dressed) |
|  |  | - circulatory problems due to cardiopulmonary limitations (n=2)   (dizziness when bending over, etc.) |
|  |  | - problems in executing object control and locomotor skills due to perceptual problems (n=2)   (problems with placing the foot when climbing stairs; positioning the feet for a firm stand; balance problems, etc.) |
|  |  | - problems in executing object control skills due to visual limitations (n=1)   (problems grasping an object due to double vision) |
|  |  | - coordination problems |
|  |  | - problems in coordinating the upper extremities (n=41)   (problems in coordinating the hands when catching a ball with both hands; problems in controlling the racket or hitting the shuttlecock when playing shuttlecock or badminton; problems working with a pen with adequate force regulation, etc.) |
|  |  | - problems in coordinating the lower extremities (n=17)   (stumbling while running; problems hitting a ball with a foot, etc.) |
|  |  | - problems in coordinating the upper and lower extremities as well as the trunk (several body parts) (n=61)   (problem building body tension; balance problems when walking or riding a bicycle; problems in coordinating the arms and legs when swimming, etc.) |
|  |  | - slow reaction (n=3)   (problems in executing motor skills due to slow reactions) |
|  |  | - deficit in physical strength |
|  |  | - deficits in strength development of the upper extremities and trunk (n=2)   (insufficient hand strength to hold a racket when playing shuttlecock; insufficient arm strength to hold the body weight when climbing, etc.) |
|  |  | - deficits in strength development of the lower extremities (n=1)   (insufficient leg strength to move the body upwards or downwards when walking stairs) |
|  | **psycho-social problems** | - fear of a ball (n=1)   (fear of catching a ball) |
|  |  | - low self-esteem (n=2)   (lack of self-confidence when climbing) |
|  |  | - fear of heights (n=2)   (fear of heights when balancing) |
|  | **cognitive problems** | - problems in understanding a movement task (n=14)   (problems in understanding movement tasks, rules of games, or verbal instructions) |
|  |  | - problems in planning and executing an action (n=12)   (difficulties in executing a motor action targetly or positioning different body parts following a verbal instruction) |
|  |  | - perceptual problems (n=5)   (problems in recognizing and evaluating obstacles; difficulties in using the appropriate shoe on the correct foot; impairment of body schema) |
|  |  | - problems with motor imagery (n=2)   (difficulties in executing a motor action or positioning different body parts (in a targeted way) following a verbal instruction) |
|  |  | - problems in anticipating movement sequences (n=1) |
|  |  | - difficulties in recognizing the affordance of an object (n=1) |
|  |  | - problems in automatizing movement sequences (n=4) |
|  |  | - attention problems (n=4)   (lack of attention when performing skills requiring precision, such as tying shoes, etc.) |
| **environmental problems** | | - functional and movement limitation through assistive devices (n=7)   (problems when bending due to a corset; difficulties when walking or running due to orthoses, etc.) |
|  |  | - problems in executing locomotor skills due to uneven terrain (n=3)   (stumbling or falling while walking; unsteady gait) |
|  |  | - problems in object control skills due to background noises (n=1)   (problems grasping a glass) |
| **task-related problems** | | - problems in executing complex movement tasks (n=6)   (difficulties in steering or braking with the arms / hands, and pedaling with the legs simultaneously; problems focusing on several things, such as rules, fellow players, game object, in group games, etc.) |
|  |  | - problems in executing complex movements (control, locomotion, manipulation) (n=10)   (problems performing the leg movement while cycling; problems coordinating the arms and legs when swimming; problems combining several partial movements into one target movement in gymnastics, etc.) |
|  |  | - problems in executing movement tasks (object control) demanding high precision (n=11) |
|  |  | - problems in executing movement tasks (locomotion) demanding high speeds (n=2) |

**Table 4.** *Tabular presentation of the thematic main code d with subcodes. Numbers in brackets indicate the number of codings.*

| **factors influencing movement behavior and movement desires of students with ID** | | | |
| --- | --- | --- | --- |
|  | | **areas of influence** | |
| **characteristic features of influencing factors** | | **motor behavior** | **movement desires** |
| **individual factors** | **physical-motor factors** | - level of physical-motor development (n=3) - physical impairment / illness (n=25) - body weight (n=5) - perception (organic) (n=13) - movement coordination (n=36) - balance (n=12) - reaction ability (n=2) - flexibility (n=5) - strength (n=6) - endurance (n=1) | - level of physical-motor development (n=1) - physical impairment (n=5) |
|  | **psycho-social factors** | - self-awareness (n=1) - self-esteem (n=4) - risk-awarenesss (n=1) - insight to perform physical activity (n=2) - motivation (n=12) - emotional state (n=2) - fear of heights (n=2) - fear of a ball (n=1) - practice time (n=5) - behavioral problems (abnormalities) (n=2) | - sense of belonging (n=2) - independence and self-determination (n=5) - urge to discover (n=1) - adventurous spirit (n=1) - need for recognition (n=2) - career aspirations (n=3) - motivation (n=6) - willpower (n=2) |
|  | **cognitive factors** | - level of cognitive development (n=8) - comprehending the movement task (n=14) - action planning and execution (n=12) - perception (n=13) - motor imagery (n=2) - affordances (n=3) - anticipation of movement sequences (n=1) - automation of movement sequences (n=4) - attention (n=4) | - level of cognitive development (n=7) - perception (n=2) |
| **environmental factors** | | - environmental conditions (playgrounds, uneven terrain / surface etc.) (n=9) - spatial conditions (n=4) - background noises (n=2) - social support (n=16) - social environment (n=19) - early promotion (n=2) - therapeutic aids (n=13) - equipment / material (n=5) - provision of assistive devices (n=8) - medical surgical procedures (n=1) - physical activity events (n=15) - competence / action of the teacher (n=7) - promotion of independence by parents or teachers (n=8) - teacher-student relationship (n=1) - number of supervising staff (n=1) - experiences (n=1) | - social environment (n=8) - social support (n=3) - media (n=3) - equipment / material (n=2) - physical activity events (n=2) - barrier-free access to materials (n=1) - availability of materials (n=1) |
| **task-related factors** | | - organization of the physical activity task (group activities) (n=2) - appeal of the physical activity task (n=3) - speed of movement execution (n=2) - precision of movement execution (n=3) - complexity of the movement task (multiple tasks) (n=8) - complexity of the movement (control, locomotion, manipulation) (n=9) | - usefulness of the physical activity task (n=1) - organization of the physical activity task (group activities) (n=2) - appeal of the physical activity task (n=2) - efficacy of the physical activity task (n=1) - structure and format of the physical activity task (competitive spirit) (n=1) |

**Table 5.** *Tabular presentation of the thematic main code e with subcodes. Numbers in brackets indicates the number of codings.*

| **content related to physical-motor and therapeutic activities in schools for students with ID** | | | |
| --- | --- | --- | --- |
| **offerings within physical-motor and therapeutic interventions** | | | |
| **sports and movement activities (courses)** | | **forms of therapy** | |
| - various sports and movement activities within physical education classes (n=12) - topic-and-interest-specific sports and movement activities (swimming group, running group, etc.) (n=9) - need-specific sports and movement activities (sports for advanced, wheelchair sports, etc.) (n=4) - inclusive (cooperative) movement activities (sports clubs, circus) (n=1) | | - physiotherapy (n=8) - occupational therapy (n=5) - speech therapy (n=1) - riding therapy (n=5) | |
| **measures (topics) within physical-motor and therapeutic interventions** | | | |
| **measures (topics) within physical-motor interventions** | | **measures (topics) within therapeutic interventions** | |
| - general movement coordination and physical conditioning / fitness - general sports-related / fundamental motor skills - gross motor skills - hopping (n=2) - roll off (a mat) (n=1) - forms of locomotion (walking, running, crawling) (n=10) - overcoming obstacles (n=1) - moving in a landscape (n=3) - climbing (n=9) - fine motor skills - manipulating objects (n=1) - bouncing (a ball) (n=1) - rolling (a ball) (n=1) - cardio / endurance training - training on a crosstrainer (n=1) - training on a cycle ergometer (n=3) - walking (n=4) - Nordic Walking (n=4) - running (n=2) - balance / sensorimotor training (n=4) - strength and flexibility training (Yoga, Pilates, strength training) (n=3) - motor skills with moveable sports equipment (rolling, riding, gliding) - moving with a roller board (n=3) - roller skating / inline skating (n=1) - riding a bicycle (n=2) - driving with three or four-wheeled vehicles (trike, tricycle, bike etc.) (n=2) - sliding (n=3) - sliding / gliding with carpet tiles (n=2) - sliding / gliding with mats (n=1) - cross-country skiing (n=3) - motor skills with gymnastics equipment - trampolining (n=12) - swinging (n=2) - motor skills in water (n=8) - motor skills related to wrestling (n=2) - motor skills related to dance, artistic creation, and performance (n=2) - sports games - major games (team sports) - hockey (n=1) - soccer / wheelchair soccer (n=7) - basketball / wheelchair basketball (n=4) - handball (n=2) - small group-games - ball games (n=3) - chasing games (n=1) - running games (n=2) - water games (n=1) - experiential education (n=2) | | - respiratory therapy (n=1) - gait training (n=6) - mobility training (n=1) - vibration training (n=2) - provision of assistive devices (n=4) - oral and orofacial therapy (n=1) - massage therapy (n=1) - promotion of basic and instrumental activities of daily living (ADLs) - basic activities of daily living - eating and drinking (n=1) - toileting (n=1) - dressing and undressing (n=1) - bathing / grooming (n=1) - climbing stairs (n=1) - overcoming obstacles (n=1) - transfers (wheelchair transfers) (n=1) - instrumental activities of daily living - meal preparation (n=1) - cleaning (n=1) - cutting the gras (n=1) - pushing a wheelbarrow (n=1) - trimming hedges (n=1) - setting the table (n=1) - transporting (n=1) | |
| **approaches used within physical-motor and therapeutic interventions** | | | |
| - Bobath concept (n=1) - Feldenkrais method (n=1) - Castillo Morales concept (n=1) - Shiatsu (n=1) - psychomotor development (n=1) | | | |
| **organization of physical-motor and therapeutic interventions** | | | |
| **time-specific aspects** | **content- and exercise-specific, and organizational aspects** | **organization and social format** | **spatial aspects (facilities, rooms)** |
| - movement activities during the school hours (physical education classes, class-intern activities) (n=6) - movement activities during school breaks (n=4) - movement activities during elective periods (n=1) | - movement landscapes (n=4) - circuit training (n=1) | - individual support / individual therapy (n=5) - group-based support / group-based therapy (n=3) | - sports hall (n=5) - swimming pool (n=3) - classroom (n=2) - specialist classrooms (trampoline room, etc.) (n=7) - school grounds (schoolyard, sports field) (n=8) - playgrounds (n=9) |

**Table 6.** *Tabular presentation of the thematic main code f with subcodes. Numbers in brackets indicate the number of codings.*

| **teaching approaches within sports, movement, and therapeutic activities in schools for students with ID** | | | | | | |
| --- | --- | --- | --- | --- | --- | --- |
| **goal-specific aspects** | **content-, exercise-, and task-specific aspects** | **time-specific aspects** | **structure-and process-specific aspects** | **action-specific aspects (general methodological principles and approaches)** | **social format** | **learning environment** |
| - general objectives - promotion of enjoyment of movement (n=8) - promotion of independence (n=2) - teaching of the relevance of a movement task (n=1) - specific objectives - specific / concrete objectives (n=1) - measurable objectives (n=2) - realistic objectives (n=1) | - selectable movement tasks within a unit (n=2) - goal-oriented movement tasks (n=2) - relevant movement tasks (n=3) - attractive movement tasks (n=4) - interest-oriented movement tasks (n=3) - effective movement tasks (n=2) - promising movement tasks (n=5) - physical activities during breaks (n=1) - needs-oriented physical activity courses (n=4) - interest-specific physical activity courses (n=2) - in-class movement activities (n=1) - inclusive (cooperative) activities with clubs (n=1) | - temporally individualized movement tasks within a unit (n=1) - regular practice (n=8) - optimal length of a section and changes of content within a unit (n=1) | - clear structure of the teaching unit (n=5) - presentation of the structure of the teaching unit (at the beginning) (n=1) - integration of therapeutic measures in the everyday school life (n=1) - instruction through lead teacher (n=2) - supervision of students by supervising teachers (n=4) | - analysis of the student as a fundamental basis for action in practice (n=4) - application of general laws, rules, or principles of physical training (n=1) - individualized approach (n=19) - gradual methodological approach (n=8) - interdisciplinary / multi-disciplinary approach (n=2) - adaptation of the movement task to the situational conditions (n=1) - flexible scheduling of breaks (n=2) - rewards to increase motivation (n=2) - brief verbal instructions (n=1) - demonstrations (n=5) - feedbacks (n=2) - reflections (n=1) - visualization tools (n=5) - medical aids (n=6) - general assistance or support (n=4) - teaching / learning aids (materials, music) (n=10) - playful promotion (n=1) - competitions to promote a sense of achievement (n=1) - active participation of teachers (n=2) - teachers as role models for movement and motivators (n=3) - exchange between parents and teachers (n=1) - patience as an important feature of teachers (n=2) - Bobath concept (n=1) - Feldenkrais method (n=1) - Castillo Morales concept (n=1) - Shiatsu (n=1) - psychomotor development (n=1) | - optimal social format (n=1) - group-specific movement tasks (n=1) | - quiet environment (n=1) - environment with other people (n=1) - optimal spatial environment (n=1) |

# Coded text segments with assigned codes

**Table 7.** *Examples of coded text passages and their corresponding codes.*

| text passages | code | application of the code / coding rule |
| --- | --- | --- |
| „fine motor problems“,  „overweight“,  „low self-esteem“,  “autism”,  „cognitive developmental delay / impairment“ | general characteristics of children and adolescents with ID | Text segments that describe general characteristics (properties – special features, abnormalities, similarities, differences) related to the body, motor function, psychological and social behavior, and cognition in children and adolescents with cognitive / intellectual impairments are assigned to this code.  Differentiation from this code: A text segment that for example describes the activity "writing" is coded with the code "motor skills ..." or subcodes of this code, or with the code "problems related to movement behavior" or subcodes of this code, but not with this code or subcodes of this code. |
| “... my primary goal is ... that the child enjoys physical activity ..." (P15, physiotherapist),  “... you simply have to focus on having fun, not on training success, ... That's the most important thing because otherwise they don't move a lot. And if you can get them to enjoy moving and have a good time in physical education, ... then you've already achieved something. And if you can ... get them to do that now and then in their free time, or maybe do a bit of inline skating or cycling, then you've achieved even more." | promotion of the enjoyment of movement | Text segments containing teachers’ statements about practices, or observations that emphasize making movement experiences enjoyable, and encouraging students to engage in physical activity with positive emotions are assigned to this code. |
| "... it's important to simply consider different goals for each student and, … to tailor the activity to each student. I think that's the key for everything, whether it's physical education or movement in the classroom ..." | individualized approach | Text segments, including teachers’ statements about approaches to promote students’ movement behavior adapted to their individual characteristics or needs, are assigned to this code. |
| "All the movement steps that we can teach a non-physically or a non-mentally disabled person in a single movement sequence, we have to divide into very ... small steps for people with mental and physical disabilities. Always maintain an external focus; always keep in mind that "moving object" to "moving object" doesn't work. So, it's best to practice from a standing position, then while walking, then while running ... These (aspects) are absolutely important - (to consider) very ... small steps, and then putting the movements together." | gradual methodological approach | Text segments including teachers’ statements about approaches to promote students’ movement behavior based on a stepwise or progressive method are assigned to this code. |
